# Motor Competence and Attainment of Global Physical Activity Guidelines among a Statewide Sample of Preschoolers

**DOI:** 10.3390/ijerph17228546

**Published:** 2020-11-18

**Authors:** Anthony Slaton, Alysse J. Kowalski, Amy Zemanick, Ann Pulling Kuhn, Erin R. Hager, Maureen M. Black

**Affiliations:** 1 Department of Pediatrics, School of Medicine, University of Maryland, Baltimore, MD 21201, USA; aslaton1@pride.hofstra.edu (A.S.); akowalski@som.umaryland.edu (A.J.K.); azemanick@som.umaryland.edu (A.Z.); apullingkuhn@som.umaryland.edu (A.P.K.); ehager@som.umaryland.edu (E.R.H.); 2 Donald and Barbara Zucker School of Medicine at Hofstra/Northwell, Hofstra University, Hempstead, NY 11549, USA; 3 Department of Epidemiology and Public Health, School of Medicine, University of Maryland, Baltimore, MD 21201, USA; 4 RTI International, Research Triangle Park, Durham, NC 27709, USA

**Keywords:** preschooler, physical activity, motor competence

## Abstract

Global physical activity guidelines for preschoolers include 60 min of moderate-to-vigorous physical activity (MVPA) daily. This study, based on the developmental model of motor skill competence, examines how motor competence relates to preschoolers’ likelihood of meeting global guidelines using ankle accelerometry. We measured physical activity using 24-h ankle-placement accelerometry (Actical) for at least two consecutive days (87% with six-seven days), motor competence using the Test of Gross Motor Development-2 (TGMD-2), and BMI-for-age z-scores (BMIz) using anthropometry and age- and sex-specific CDC norms. Caregivers provided demographic characteristics of children’s age, sex, and race. We used multivariable logistic regression to examine how motor competence, BMIz weight status, and demographic characteristics related to meeting global physical activity guidelines. The sample included 588 preschoolers, age 3–5 years; 55% male; 60% white; and 28% overweight/obese; 75% attained the recommended 60 min of MVPA per day. The odds of meeting MVPA guidelines were associated with higher gross motor quotient, higher object control scores, sex (male), age (older), and race (white), but not with BMIz weight status. Findings support the use of 24-h ankle accelerometry among preschoolers and are consistent with the developmental model of motor competence applied to preschoolers, whereby object control competence relates positively to attaining global physical activity guidelines.

## 1. Introduction

Excess weight gain in preschool-age children (3–5 years of age) is generating widespread interest due to its association with obesity-related comorbidities later in life [1,2]. In the United States, 13.9% of children ages 2–5 have obesity [3]. Identifying the factors that underlie the current obesity epidemic is a public health priority. Limited physical activity is regarded as an early risk factor for obesity [4,5]. As such, obesity prevention efforts that target physical activity in young children have the potential to reduce children’s lifelong obesity risk. Adequate physical activity during early childhood not only reduces the risk of obesity but also plays an important role in social development, cognition, and emotional well-being [4,5]. The promotion of physical activity in preschoolers requires a thorough understanding of the multiple factors that influence children’s movement behavior, including motor competence, weight status, and demographic variables, including sex, age, and race/ethnicity.

The developmental model of motor skill competence introduced by Stodden and colleagues suggests that there is a synergistic relationship between motor skill competence and physical activity during childhood, with advances in one impacting the other [6]. Motor skill competence is defined as the development of object control skills (e.g., kicking, throwing) and locomotor skills (e.g., running, skipping) [6]. A recent review found that preschoolers who demonstrated mastery of locomotor and object control motor skills were more likely to be physically active [7]. Basic motor skills developed in early childhood provide the foundation for more complex movements required for participation in sports and other organized activities later in adolescence [8]. The importance of motor competence in physical activity is reflected in the National Association for Sport and Physical Education’s (NASPE) Active Start guidelines which explicitly state that motor skill development should be an integral component of early childhood education [9]. Despite these guidelines, recent evidence suggests that the heterogeneity in motor competence may reflect differences in children’s, sex, age, race/ethnicity and weight status [10,11,12,13,14], indicating a need to further explore these relationships.

A key issue with much of the literature regarding physical activity in young children is that it relies on data collected in structured environments, such as childcare centers, where a caregiver or teacher directs children’s daily activity. In a sample of same-sex twin pairs, Fisher and colleagues found that children’s environment was the most significant predictor of physical activity [11]. As such, there is a need for studies that measure physical activity throughout an entire 24-h period to elucidate individual differences that potentially influence motor development. The current study aims to assess 24-h physical activity among preschoolers across multiple contexts (preschool and home) that include structured and unstructured physical activity. This is accomplished by using ankle accelerometers to track children’s movement for a 7-day period. Placement of the accelerometer on the ankle allows investigators to examine relations among motor competence, physical activity, environmental, and child characteristics throughout multiple activities.

The World Health Organization and the NAPSE recommend that preschoolers engage in at least 60 min of moderate-to-vigorous physical activity (MVPA) per day [9,10]. Despite these guidelines, the proportion of children engaging in 60 min of MVPA per day varies greatly across studies depending on the measurement method used. For example, Guinhouya and colleagues found that the proportion of children engaging in 30 min of MVPA varied from 34.8% to 100% depending on the methodology selected [15]. Objective monitoring of physical activity using accelerometry is the most accurate method to measure physical activity. Still, there is little consensus on accelerometer cut points most acceptable to signify MVPA among preschoolers [16]. The use of different cut points could have significant impacts on reporting as the selection of cut point thresholds influences the proportion of children who meet MVPA guidelines [15,17,18]. Consequently, physical activity measurement among preschoolers is not well understood. The purpose of this study is to examine how motor competence and demographic characteristics relate to preschoolers’ likelihood of engaging in 60 min of MVPA per day, assessed via 24-h ankle accelerometry.

## 2. Materials and Methods 

As a preliminary step, we conducted a search of cut points used with Actical accelerometers. We identified 24 papers: 21 used right hip placement, two used ankle placement, and one used wrist placement. We could not identify a current threshold for ages 3–5 on ankle placement, therefore we applied the ankle thresholds validated by indirect calorimetry for ages 12–36 [19]. All children in the validation sample walked independently, the mean age was 24.5 months (range 14.7–35.5), 58.3% were males, and they engaged in six specific activities: (1) watching TV (sitting), (2) listening to a book (sitting), (3) table games (standing), (4) imaginary play (walking), (5) ball games (running), and (6) chase (running) [19]. 

### 2.1. Participants

Data were collected from children in 54 childcare centers in ten Maryland counties as part of a larger three-arm cluster randomized controlled childhood obesity prevention trial: Creating Healthy Habits Among Maryland Preschoolers (CHAMP) [20]. Childcare centers that served low- and middle-income communities in urban, semi-urban and rural areas were eligible if they accepted childcare vouchers, participated in the Child and Adult Care Food Assistance Program, or cost less than $300/week per child. Head Start centers were excluded because many were already engaging in health promoting activities. Children were eligible if they were between 3–5 years of age, attended childcare at least 3 days per week, were English-speaking, intended to be enrolled in the center through the spring, and were without developmental delays that interfered with assessment. The current study examined data collected at baseline evaluations prior to intervention. Evaluations occurred on site at the childcare centers. All parents gave their informed consent for their child’s inclusion before they participated in the study. The study was conducted in accordance with the Declaration of Helsinki, approved by the Ethics Committee of the University of Maryland School of Medicine (HP-00065933), and registered at https://clinicaltrials.gov/ct2/show/NCT03111264.

### 2.2. Measures

#### 2.2.1. Anthropometry and Demographics 

Height was measured with a portable stadiometer (Shorr Productions, Olney, MD, USA) to the nearest 1 cm. Weight was measured with TANITA BWB-800 (TANITA, Tokyo, Japan) digital scales to the nearest 0.1 kg. Two independent measurements were taken and repeated if the two initial measures were not identical. We used height (meters, m) and weight (kilograms, kg) measurements to calculate BMI (weight/height^2^, kg/m^2^) and BMI-for-age z-scores (BMIz), based on the age- and sex-specific 2000 CDC norms [21]. Children with BMIz scores: z < −1, −1 ≤ z ≤ 1, and z > 1 were considered to be underweight, healthy weight, and overweight/obese, respectively. Caregivers reported demographic characteristics of the child in an online survey administered through Qualtrics. We coded demographic characteristics as: sex (0 male, 1 female) and race (0 white, 1 other). 

#### 2.2.2. Physical Activity 

Children’s physical activity was assessed via 24-h accelerometers (Actical, Phillips Respironics, Inc.). At the childcare center, accelerometers were secured to the lateral malleolus of each child’s non-dominant ankle, under socks, using a non-removable band. Children wore the accelerometer for 7 days. Protocol and data handling were based on prior studies that used the device to measure physical activity and sleep in young children [19]. Data were collected by the accelerometers in 15-s epochs. We applied cut points to calculate time spent in MVPA −550 counts per 15 s [22,23]. We retained data for children with at least two days of valid data (87% had valid data for at least six days). Preschoolers engaging in at least 60 min of MVPA per day were coded as having met MVPA guidelines (0 did not meet guidelines, 1 met guidelines) [9,10]. 

#### 2.2.3. Gross Motor Skill 

We administered the Test of Gross Motor Development-2 (TGMD-2) to assess children’s gross motor skill development. The TGMD-2 is comprised of two subtests – object control and locomotor [24]. The object control subtest measured children’s mastery of skills such as throwing and catching a ball. The locomotor subtest measured children’s mastery of fluid coordinated movements from one point to another. Trained research assistants observed each child individually perform a series of items (movements) such as throwing a ball, skipping, running, and hopping. Each item was performed twice and assigned a score of “1” for accurate completion or “0” for non-completion. Items were summed to produce raw scores. 

We selected TGMD-2 because it provides a granular assessment of the child’s motor development-based upon a discrete set of criteria for each skill. For example, locomotor development is assessed by observing a child run, gallop, hop, leap, slide, and jump horizontally. Running, for instance, is then further deconstructed into a specific set of performance criteria: moving arms in opposition to legs, striding with both feet off the ground for a brief period of time, narrow foot placement, and stepping with the non-support leg bent at 90 degrees. The individual performance criteria are then scored 0 or 1 for completion, while the overall subtest score—used in the linear regression—is a composite of the entire set of items for the various movements. This process provides a quantitative assessment of the child’s motor development on a scale of 1–20 after adjustments for sex and age.

The TGMD has good to excellent inter-rater reliability [24]. In our sample, the mean inter-class correlation coefficient (ICC) was 0.98. Gross motor skill development is influenced by biological maturation, with male sex associated with better development [25]. Some evidence suggests that there are pre-maturational biological differences favoring males in skills such as throwing and striking [26]. To adjust for potential biological differences, we converted raw scores to age- and sex-adjusted standard scores on the TGMD-2, as recommended. We combined the standardized subtest scores to create an overall performance score, the Gross Motor Quotient (GMQ), which is a valid and reliable measure of children’s current gross motor development. GMQ scores <17, 17–23, and 24–40 reflect poor to below average, average, and above average to superior gross motor development, respectively [27,28]. 

### 2.3. Statistical Analysis

We included participants with gross motor development and physical activity measures in the analysis. We characterized the sample using frequencies for categorical measures and mean ± standard deviation for continuous measures. 

Using the ankle cut-point of ≥550 counts per 15 s to signify MVPA, we calculated average daily minutes spent in MVPA for each child [22]. Based on prior literature involving young children, we considered age, sex, race, BMIz weight status, object control, locomotor, and GMQ as a priori predictors of time spent in MVPA. We conducted simple linear regression with minutes of MVPA per day for each predictor. Predictors that were significantly associated with minutes of MVPA per day in unadjusted models were included in a multivariable linear regression model. Using unadjusted logistic regression, we examined associations of demographic characteristics and motor competence with meeting MVPA guidelines. Significant predictors were included in multivariable models. We conducted statistical analyses using STATA (version 14) and considered *p* < 0.05 statistically significant. 

## 3. Results

Child demographic characteristics and performance on the TGMD-2 test are displayed in Table 1. Of the 588 preschoolers with gross motor development and physical activity measures, 55% were male, 60% identified as white, and 28% had overweight or obesity. On average, preschoolers engaged in 76.5 min of MVPA each day. Over half (54%) of preschoolers scored above average for their age on the TGMD-2 while 8% performed below average. Almost three-quarters of preschoolers (*n* = 439/588, 74.7%) reached recommended levels of MVPA per day when ankle placement cut points were applied. 

Linear regression results are displayed in Table 2. Preschoolers who were older, male, and white spent more time in MVPA compared to younger, female, and non-white preschoolers in unadjusted and adjusted models; and weight status was not associated with time spent in MVPA. Object control, locomotor scores, and GMQ were not associated with minutes of MVPA/day in unadjusted or adjusted linear models. In multivariable logistic regression, increased GMQ was associated with slightly increased odds of meeting MVPA guidelines (OR = 1.05, 95% CI: 1.00, 1.09) (Table 3). This was driven by object control which was positively associated with meeting MVPA guidelines (OR = 1.10, 95% CI: 1.01, 1.19). Locomotor was not associated with meeting MVPA guidelines in unadjusted or adjusted models. The odds of meeting MVPA guidelines were twice as high for males compared to females and twice as high for white preschoolers compared to non-white preschoolers. Weight status was not associated with meeting MVPA guidelines. 

## 4. Discussion

Guided by the development model of motor skill competence and using objectively measured 24-h physical activity data from ankle placement, this study had three primary findings. First, 75% of preschoolers met the criterion of 60 min of daily MVPA. Second, object control skill competence, but not locomotor, was significantly associated with meeting the criterion of 60 min of daily MVPA. Third, sex (male), older age, and white race, but not weight status, were significant predictors of 60 min of daily MVPA. 

To compare the proportion of preschoolers in our study meeting the global guidelines of 60 min of daily MVPA, we reviewed the Canadian 24-Hour Movement Guidelines for the Early Years that found nearly all (62–84%) of Canadian preschoolers reached recommended levels of MVPA [29,30]. We also referenced a recent report from the International Children’s Accelerometry Database (ICAD) [31]. The analysis included 1052 preschoolers (ages 3 and 4 years) from six studies in four high-income countries (Canada, Australia, United States, and United Kingdom), using waist-worn, uniaxial Actigraph accelerometers. Overall, 78.8% of children in the ICAD study met the criterion of 60 min of daily MVPA. Using cut points established by Pate [32], at least three studies examined the prevalence of preschoolers meeting the criterion of 60 min of MVPA. In one study, the prevalence varied from 77.6% (weekends) to 93.5% (weekdays) [33]. In a second study, the prevalence was 52.9% among girls and 65.6% among boys [17]. In a third study conducted among preschoolers having a BMI above the 85th percentile who were referred for treatment for overweight/obesity, the prevalence was 80% [34]. Thus, our findings of 75% were congruent with the literature.

The finding that object control skills, including ball handling, positively predicted preschoolers’ attainment of 60-min of daily MVPA is consistent with the developmental model of motor competence and with other studies that have found positive relationships between motor competence and physical activity [6,35]. Among preschoolers, object control represents a basic skill that children need to be physically engaged in preschool activities, such as ball handling [25]. Beyond the biological maturation required to learn basic motor skills, opportunities to learn and practice motor skills are needed to advance motor skills [25]. The developmental mode of motor skill competence, informed by Ecological Systems Theory [36] and Achievement Goal Theory [37], posits that the environmental context influences opportunities to gain object control skills and to increase children’s confidence and perceptions of their object control skills. Based on the developmental model of motor competence, children’s perception of their motor skills mediates the association between object control skills and physical activity, such that gaining object control skills enhances self-perception and enjoyment, which increase the likelihood of seeking out opportunities for subsequent physical activity.

The relation between motor competence and physical activity among preschoolers has been mixed. In a study among preschool-age children in low-income communities in Brazil, 72% of girls and 84% of boys achieved 60 min of daily MVPA, but meeting physical activity guidelines was not associated with overall motor competence [38]. Another study found a relationship between MVPA and locomotor skills, but not object control skills [31]. A third study found that male object control skills were associated with physical activity, while female locomotor skills were associated with physical activity [35]. Finally, a meta-analysis reported consistent relations among object control and MVPA [39]. These findings suggest that among preschoolers, exposure to specific activities and skills may contribute to increases in motor competence, particularly object control, which in turn is associated with increases in physical activity. This pattern is consistent with the synergism proposed by the developmental model of motor competence [6].

Our initial set of analyses were consistent with previous literature that MVPA was greater among male preschoolers compared to females [35] and among older compared to younger preschoolers [40]. There are at least two possible explanations for these findings. First, older age and male sex have been consistently related to object control skills [41], suggesting that age and sex differences in preschoolers’ MVPA may be associated with differences in object control skills. Second, when environmental and sociocultural factors are considered, preschoolers’ physical activity is largely associated with the availability of open spaces for play, and family involvement, encouragement, and expectations related to physical activity, which is often higher for male compared to female preschoolers [42,43]. Although at least one other study has found racial differences related to physical activity among preschoolers, most reviews have not reported racial differences in MVPA among preschoolers, particularly when environmental and sociocultural variables were considered [40].

Our finding that preschoolers’ weight status was not related to meeting the criterion of 60 min of MVPA daily is consistent with findings from a systematic review of physical activity [44] and a meta-analysis of motor competence [25] that among preschoolers, BMIz was not related to either. However, a systematic review showed that preschool interventions that promote physical activity are effective in improving children’s weight status, illustrating the beneficial effects of incorporating physical activity into obesity prevention [45].

Overall, these findings suggest that limited physical activity in early childhood could exacerbate health disparities in obesity-related comorbidities found in adult populations. Although all preschoolers should have opportunities to advance their motor skill competence and engage in physical activity, younger, female preschoolers may benefit from additional initiatives. Providing preschoolers with opportunities to engage in and improve their motor competencies is important for participating in healthy physical activity patterns throughout life [8]. 

This study yields several recommendations for future investigations. First, gross motor competency is a general term that includes multiple proficiencies, including object control, locomotor skill, and coordination. Clarifying the factors associated with specific types of motor competency would reduce confusion. Second, as suggested by the developmental model of motor competence, future research among preschoolers should examine the mechanisms linking environmental opportunities and preschoolers’ perception of their motor competence to motor competence and physical activity. Third, future research among preschoolers should examine associations among demographic, environmental, and sociocultural factors related to motor competence and physical activity longitudinally. Research can also examine underlying reasons for differences in motor competencies such as the type of sport or activity participation or physical education curricula, as well as examine what type of physical activity is important for motor development [46]. There have been at least two reviews of interventions to promote physical activity among preschoolers, which is particularly relevant with increases in preschool enrollment [47,48]. One recommended that interventions for preschool children be theory-driven, involve children and parents, and include a structured activity delivered by experts [47]. Another recommended that preschoolers need developmentally appropriate instruction in motor competence, as well as opportunities to practice and develop a positive self-perception of their motor competencies [48]. Both endorsed the importance of the preschool period as an ideal time to help children develop motor competence and an affinity for physical activity.

### Strengths and Limitations 

There were several strengths and limitations to this study. The focus on the association between motor skill competence and physical activity among preschoolers represents an understudied topic during a period when young children are establishing habits that can influence their health and well-being across the life-course. Direct measurement of MVPA through accelerometry and motor competence through the TGMD-2 avoids self-report biases. The inclusion of statewide data from preschoolers in low- and middle-income families from urban, semi-urban, and rural areas is wide representation. Although ankle accelerometry allows for 24-h continuous wear and reduces participant burden, it introduces the burden of wearing a device, may not capture activity conducted while seated, and may over-estimate fidgeting involving the ankle. A validation study should be conducted among preschoolers. Additional limitations include a cross-sectional design, thus not permitting the examination of the temporality of motor skill competence and physical activity, and the lack of data on important covariates, including household family socioeconomic variables [49]. 

## 5. Conclusions

Motor skill competence and physical activity play critical roles in early childhood in promoting cognition, school readiness, and habits that facilitate health and well-being throughout life [5]. The benefits associated with adequate physical activity levels can be seen across a broad spectrum of physical, social, and cognitive indicators in children [50]. 

Interventions that promote physical activity habits have been effective in preventing obesity among preschoolers [45], illustrating the relevance of understanding the mechanisms that underpin physical activity among preschoolers. Overall, our study provides insight into the association between motor competence and physical activity, the role of ankle accelerometry in providing 24-h measurement, and the need to promote interventions that target improving object control skills in preschoolers.

## Figures and Tables

**Table 1:** Descriptive characteristics of CHAMP participants with physical activity and gross motor development measures (*N* = 588) ^a^.

Demographic Characteristic	*N*	*n*	%
Age	588		
3 years-old		257	44%
4 years-old		290	49%
5 years-old		41	7%
Sex	588		
Male		321	55%
Female		267	45%
Race	538		
White		321	60%
Other		217	40%
BMIz weight status	561		
Underweight (z < −1)		49	9%
Healthy weight (−1 ≤ z ≤ 1)		356	63%
Overweight or obese (z > 1)		156	28%
MVPA		Minutes	SD
Average per day	588	76.5	29.3
Motor Competence		*N*	%
TGMD-2: Gross Motor Quotient	588		
Above Average–Superior (24–40)		316	54%
Average (17–23)		227	39%
Below Average–Very Poor (2–16)		45	8%
		Average	SD
TGMD-2: Object Control Subtest Standardized Score	588		
3 years-old		12.5	3.0
4 years-old		12.4	2.4
5 years-old		11.5	3.0
TGMD-2: Locomotor Subtest Standardized Score	588		
3 years-old		11.1	3.4
4 years-old		12.1	3.2
5 years-old		11.3	3.3

^a^ Presented as *N* (%) or mean ± SD. Abbreviations: BMIz, body mass index z-score; MVPA, moderate-to-vigorous physical activity; TGMD-2, Test of gross motor development-2.

**Table 2:** Unadjusted and adjusted linear associations of gross motor development and child demographic characteristics with mean minutes of moderate-to-vigorous physical activity per day.

Characteristic	β (95% CI) ^a^	*p*	β_adj_ (95% CI) ^b^	*p*	β_adj_ (95% CI) ^b^	*p*
Sex						
Male	11.23 (6.56, 15.9)	<0.01	11.46 (6.61, 16.3)	<0.01	11.14 (6.33, 15.95)	<0.01
Age	0.55 (0.22, 0.87)	<0.01	0.6 (0.27, 0.94)	<0.01	0.57 (0.24, 0.9)	<0.01
Race						
White	8.94 (3.96, 13.93)	<0.01	8.31 (3.43, 13.19)	<0.01	8.58 (3.72, 13.44)	<0.01
BMIz weight status						
Underweight	5.44 (−3.28, 14.17)	0.22	--		--	
Healthy weight	Ref		--		--	
Overweight	1.2 (−4.3, 6.71)	0.67	--		--	
TGMD-2						
Object Control	0.58 (−0.28, 1.45)	0.19	0.8 (−0.21, 1.81)	0.12	--	
Locomotor	0.3 (−0.42, 1.01)	0.42	−0.07 (−0.91, 0.78)	0.88	--	
Gross Motor Quotient	0.28 (−0.17, 0.74)	0.22	--		0.31 (−0.16, 0.78)	0.19

^a^ Models are unadjusted. ^b^ Models are adjusted for other characteristics in column. Abbreviations BMIz, body mass index z-score; TGMD-2, Test of gross motor development-2.

**Table 3:** Unadjusted and adjusted logistic associations of gross motor development and child demographics with meeting moderate-to-vigorous physical activity guidelines per day.

Characteristic	OR (95% CI) ^a^	*p*	OR (95% CI) ^b^	*p*	OR (95% CI) ^b^	*p*	OR (95% CI) ^b^	*p*
Sex								
Male	2.18 (1.49, 3.19)	<0.01	2.26 (1.5, 3.42)	<0.01	2.4 (1.59, 3.66)	<0.01	2.34 (1.55, 3.55)	<0.01
Age	1.06 (1.03, 1.09)	<0.01	1.07 (1.04, 1.1)	<0.01	1.07 (1.04, 1.1)	<0.01	1.06 (1.03, 1.1)	<0.01
Race								
White	2.21 (1.49, 3.28)	<0.01	2.26 (1.51, 3.41)	<0.01	2.16 (1.43, 3.28)	<0.01	2.22 (1.47, 3.35)	<0.01
BMIz weight status								
Underweight	1.07 (0.54, 2.27)	0.86	--		--		--	
Healthy weight	Ref		--		--		--	
Overweight	0.93 (0.6, 1.44)	0.73	--		--		--	
TGMD-2								
Object Control	1.08 (1.01, 1.16)	0.03	--		1.1 (1.01, 1.19)	0.04	--	
Locomotor	1.05 (0.99, 1.11)	0.11	--		1.01 (0.94, 1.08)	0.87	--	
Gross Motor Quotient	1.04 (1, 1.08)	0.03	--		--		1.05 (1, 1.09)	0.03

^a^ Models are unadjusted. ^b^ Models are adjusted for other characteristics in column. Abbreviations: OR, odds ratio; BMIz, body mass index z-score; TGMD-2, Test of gross motor development-2.
